# The clinical implication of new‐onset in‐hospital atrial fibrillation in patients with acute decompensated heart failure

**DOI:** 10.1002/joa3.12386

**Published:** 2020-07-07

**Authors:** Masashi Kamioka, Akiomi Yoshihisa, Minoru Nodera, Tomofumi Misaka, Tetsuro Yokokawa, Takashi Kaneshiro, Kazuhiko Nakazato, Takafumi Ishida, Yasuchika Takeishi

**Affiliations:** ^1^ Department of Cardiovascular Medicine Fukushima Medical University Fukushima Japan; ^2^ Department of Advanced Cardiac Therapeutics Fukushima Medical University Fukushima Japan; ^3^ Department of Arrhythmia and Cardiac Pacing Fukushima Medical University Fukushima Japan

**Keywords:** acute heart failure, atrial fibrillation after discharge, cardiac death, cerebrovascular event, new‐onset in‐hospital atrial fibrillation

## Abstract

**Background:**

To investigate the clinical implication of the temporal difference in atrial fibrillation (AF)‐onset in acute decompensated heart failure (ADHF) and its impact on post‐discharge prognosis.

**Methods:**

336 new‐onset ADHF patients without any history of AF before admission were enrolled (201 males, 63 ± 16 year‐old) and classified into two groups based on their history of AF: the Control group (No AF was detected during hospitalization, n = 278), and the In‐hos‐AF group (AF occurred during hospitalization, n = 58). Post discharge prognosis including rehospitalization due to worsening HF, cardiac death, all‐cause death and cerebrovascular event were compared.

**Results:**

Kaplan‐Meier analysis demonstrated that the incidence of rehospitalization due to HF, cardiac death, all‐cause death and cerebrovascular event in the In‐hos‐AF group was not significantly different from that in the Control group (*P* > 0.05 respectively). However, when AF recurred in the In‐hos‐AF group patients (n = 24, 41%) after discharge, the incidence of rehospitalization due to HF and cardiac deaths were higher than those without AF recurrence (*P* = 0.018 and *P* = 0.027 respectively). Cox proportional analysis revealed that AF developing after discharge was proven to be an independent risk factor for rehospitalization due to HF (HR 1.845, *P* = 0.043), cardiac death (HR 3.562, *P* = 0.013) and all‐cause deaths (HR 2.138, *P* = 0.020).

**Conclusion:**

Clinical outcomes of new‐onset in‐hospital AF patients were as good as those without AF history until AF recurrence. However, AF recurrence led to worse prognosis. Therefore, treatment for new‐onset in‐hospital AF in ADHF patients might be postponed until AF recurrence.

## Graphical abstract

Clinical outcome in the new‐onset in‐hospital AF in patients with ADHFD, compared with those who had no history of AF.

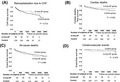

## 1 INTRODUCTION

Atrial fibrillation (AF) and chronic heart failure (CHF) are common illnesses that frequently coexist, resulting in adverse cardiovascular mortality.^1^ The prevalence of AF and CHF is increasing, and will have a severe impact on the cost of healthcare services globally.^2^, ^3^ AF has been reported to be associated with a threefold increased risk of incident heart failure (HF),^4^ and can facilitate HF via several mechanisms, such as an increase in resting heart rate‐mediated shortening of diastolic filling time, irregular ventricular response, and the loss of atrial kick.^5^ On the other hand, HF can precipitate the development of new‐onset AF.^6^ Patients at the acute worsening phase of heart failure (ADHF) sometimes develop AF, even if they have no prior history of AF. As HF goes better with the treatment, the new developing AF often terminates spontaneously and does not recur during hospital stay. To date, there is no consensus about the timing of when to initiate treatment for this kind of new‐onset AF in ADHF patients. The aim of the current study was to investigate the clinical implications of new‐onset AF during hospitalization in ADHF patients, and its clinical impact on post discharge prognosis.

## 2 METHODS

### 2.1 Study subjects

**FIGURE 1:**
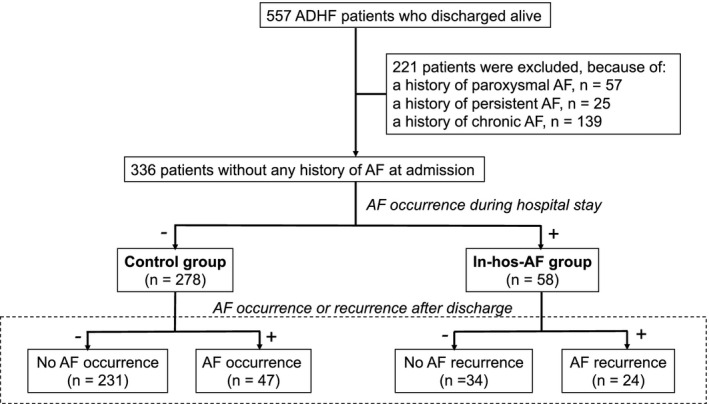
Patient flow diagram. Study patients were categorized into two groups based on the history of AF: the Control group (No AF was detected before admission and during hospitalization, n = 278), and the In‐hos‐AF group (Without history of AF, and AF occurred during hospitalization, n = 58)

This was a prospective observational study of 566 first‐time ADHF patients who were discharged from Fukushima Medical University Hospital between January 2010 and October 2012. The diagnosis of decompensated HF was made based on the HF guidelines.^7^ As shown in Figure 1, out of the 566 ADHF patients, patients who had any history of AF (n = 221) and died during hospitalization (n = 9) were excluded, and 336 without any history of AF at admission were finally enrolled. AF was identified by a 12‐lead electrocardiogram (ECG), continuous telemetry monitoring during hospitalization, and/or 24‐hour‐holter ECG before admission, during hospitalization and after discharge. Rapid AF was defined as AF with a heart rate of ≥120 bpm.^8^ AF types were defined following the current guidelines.^9^ Symptoms including palpitations, syncope, dyspnea, chest pain, dizziness, fatigue, and non‐specified symptoms were considered as AF symptoms, if these symptoms were totally different from those at the onset of heart failure. Patients who presented with these symptoms were defined as symptomatic AF patients. After discharge, all patients were followed up at 1, 3, and 6 months, then every 3 months thereafter at our university hospital or the patient's referring hospital. At each visit, a 12‐lead ECG was recorded. At each 12‐month visit, 24‐hour‐holter ECG was performed.

We classified the 336 patients into two groups based on their history of AF: the Control group (No AF was detected before admission and during hospitalization, n = 278), and the In‐hos‐AF group (without history of AF, and AF occurred during hospitalization, n = 58). NYHA functional classifications were determined at the time of discharge. Structural heart disease was diagnosed using echocardiography and coronary angiography or coronary artery imaging with computed tomography. Hypertension (HT) was defined as the use of antihypertensive drugs. Diabetes mellitus was defined as the use of antidiabetic drugs, a fasting glucose value of ≥126 mg/dL, a casual glucose value of ≥200 mg/dL, and/or a HbA1c of ≥6.5% (National Glycohemoglobin Standardization Program). Dyslipidemia (DLp) was defined as the use of cholesterol‐lowering drugs, a triglyceride value of ≥150 mg/dL, a low‐density lipoprotein cholesterol value of ≥140 mg/dL, and/or a high‐density lipoprotein cholesterol value of <40 mg/dL. Chronic kidney disease (CKD) was defined as an estimated glomerular filtration rate (GFR) of <60 mL/min/1.73 cm^2^ according to the Modification of Diet in Renal Disease formula.^10^

All patients were followed up until 2017. The prevalence of rehospitalization due to HF, cardiac deaths, all‐cause deaths and cerebrovascular events as the study endpoints between the two groups were compared. Cardiac death was classified by independent experienced cardiologists as death caused by worsened HF in accordance with the Framingham criteria, ventricular tachyarrhythmia documented by electrocardiogram or implantable devices, acute coronary syndrome, or sudden cardiac death. Sudden cardiac death, including pulseless electrical activity (PEA) and cardiac arrest, was defined as follows. At the time of sudden death, HF was controlled and no ventricular tachyarrhythmia was monitored. Although cardiopulmonary resuscitation was performed, return of spontaneous circulation could not be obtained. ECG monitor showed a heart rhythm, which should produce a pulse, but does not (PEA), or a sudden change in ECG, in which it goes flat (cardiac arrest). No contraction of the heart was detected by echocardiography. Status and date of death were obtained from the patients’ medical records. If these data were unavailable, the patient's status was ascertained by a telephone call to their patient's referring hospital physician. Cerebrovascular events were defined as stroke or systemic embolism.^11^ Stroke was defined as the sudden onset of a focal neurologic deficit in a location consistent with the territory of a major cerebral artery, including ischemic, hemorrhagic, or unspecified types. Systemic embolism was defined as an acute vascular occlusion of an extremity or organ, documented by means of imaging or surgery. Written informed consent was obtained from all study subjects. The study protocol was approved by the ethical committee of Fukushima Medical University, the investigation conforms with the principles outlined in the Declaration of Helsinki, and reporting of the study conforms to STROBE along with references to STROBE and the broader EQUATOR guidelines.^12^

### 2.2 Blood samples analysis

Venous blood was collected at hospital on the day before discharge in a state of stable compensated heart failure. Levels of B‐type natriuretic peptides (BNP) were measured.

### 2.3 Echocardiographical evaluation

Echocardiography was performed using standard techniques.^13^ Left ventricular ejection fraction (LVEF) and left atrial volume index (LAVI) were calculated. The apical 4‐ and 2‐chamber views including the entire left atrium and left ventricle were acquired, and LA and LV volumes were determined using the biplane Simpson's method. LVEF was calculated using the diastolic and systolic LV volumes. For LAVI measurement, LA volume was indexed to the body surface area.

### 2.4 Statistical analysis

A chi‐square test was used to compare dichotomous data, and the results were presented as numbers and percentages. Continuous data were analyzed using an independent‐sample *t* test, and were presented as mean ± SD. The cumulative incidence of rehospitalization due to HF, cardiac deaths, all‐cause deaths, and cerebrovascular events was evaluated using the Kaplan‐Meier method. For the analysis of the independent predictor for rehospitalization due to HF, cardiac deaths, all‐cause deaths, and cerebrovascular events, a Cox proportional hazard model was used, and expressed as a hazard ratio (HR) and confidence interval (CI). Log BNP was used in the regression model, as BNP levels were not normally distributed. In the multivariate Cox proportional hazard analysis, to prepare for potential confounding, we considered the following clinical factors, which are generally associated with the risk of study endpoints: age, gender, NYHA functional class, ischemic etiology, hypertension, diabetes, dyslipidemia, chronic kidney disease, BNP, and LVEF. These factors and univariate parameters with a *P* < .05 were included in the multivariate analysis based on the number of events. To elucidate the predictor of AF recurrence after discharge in the in‐hos‐AF group, Cox proportional hazard analysis was performed. The univariates with a *P* < .05 were included in the multivariate analysis. All analyses were performed with SPSS for Windows, version 25.0 (SPSS Inc.). All statistical tests were two‐sided, and *P* < 0.05 were considered statistically significant.

## 3 RESULTS

### 3.1 Patient characteristics

**FIGURE 2:**
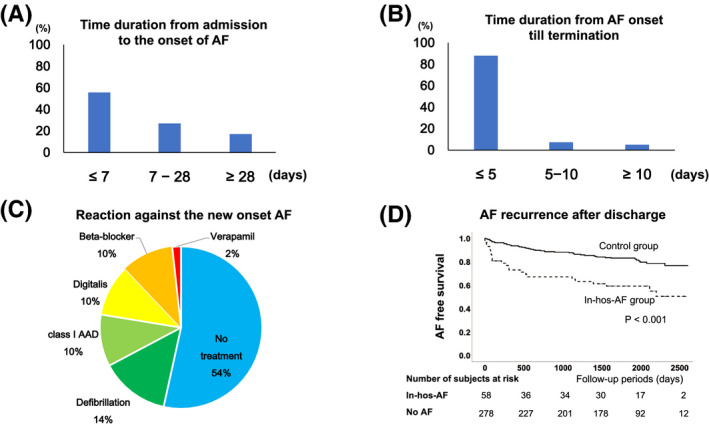
A, The time duration from admission to the onset of atrial fibrillation (AF). Fifty‐six percent of the In‐hos‐AF group patients (33 of 58 patients) experienced AF occurrence within a week after admission. B, The time duration from AF onset till termination. Fifty‐one of fifty‐eight patients of the In‐hos‐AF group patients (88%) experienced AF termination within 5 days after the onset of AF. C, The reaction against the new‐onset AF. D, Kaplan‐Meier analysis for AF recurrence after discharge

The patient characteristics between the Control and the In‐hos‐AF groups were presented in Table 1. The prevalence of CKD and NYHA functional class in the In‐hos‐AF group was significantly higher than that of the Control group. LVEF in the Control group was mildly reduced compared to that of the In‐hos‐AF group. With regard to the In‐hos‐AF group, more than half of the new‐onset AF occurred within a week (Figure 2A) and almost all of the new‐onset AF (n = 51, 88%) terminated within 5 days (Figure 2B). Therefore, 33 of 58 patients (56%) in the In‐hos‐AF groups did not undergo treatment against the new‐onset AF (Figure 2C). With regard to the AF prevalence after discharge, Kaplan‐Meier analysis demonstrated that AF prevalence in the In‐hos‐AF group was significantly higher than that of the Control group (*P* < 0.001), as shown in Figure 2D.

**TABLE 1:** Patient characteristics between the Control and the In‐hos‐AF groups

	Control group (n = 278)	In‐hos‐AF group (n = 58)	*P* value
Age (y)	63 ± 16	65 ± 14	0.265
Male (%)	58	71	0.071
BMI (kg/m^2^)	23.3 ± 4.3	23.6 ± 3.7	0.662
NYHA	1.8 ± 0.4	2.0 ± 0.3	**0.025**
CHA2DS2‐VASc score	4.3 ± 1.8	4.4 ± 1.6	0.823
Ischemic etiology (%)	27	31	0.570
Comorbidity			
HT (%)	85	86	0.747
DM (%)	43	47	0.602
DLp (%)	79	81	0.746
CKD (%)	47	62	**0.043**
Medication			
RAS inhibitor (%)	81	74	0.271
β‐blocker (%)	82	67	**0.033**
Diuretics (%)	62	69	0.310
Inotropic agents (%)	20	21	0.925
Anticoagulant (%)	42	55	0.077
DOAC (%)	1	3	0.295
VKA (%)	41	52	0.134
Lab data			
BNP	780 ± 1428	717 ± 1195	0.788
Echo data			
LVEF (%)	47 ± 16	53 ± 15	**0.037**
LAVI (m^l^/m^2^)	29 ± 24	31 ± 25	0.576

Abbreviations: BMI, body mass index; BNP, B‐type natriuretic peptide; CKD, chronic kidney disease; DLp, dyslipidemia; DM, diabetes mellitus; DOAC, direct oral anticoagulant; LAVI, left atrial volume index; LVEF, left ventricular ejection fraction; NYHA, New York Heart Association functional class; RAS, renin‐angiotensin‐aldosterone system; VKA, vitamin K antagonist. 1

### 3.2 Clinical outcome

**FIGURE 3:**
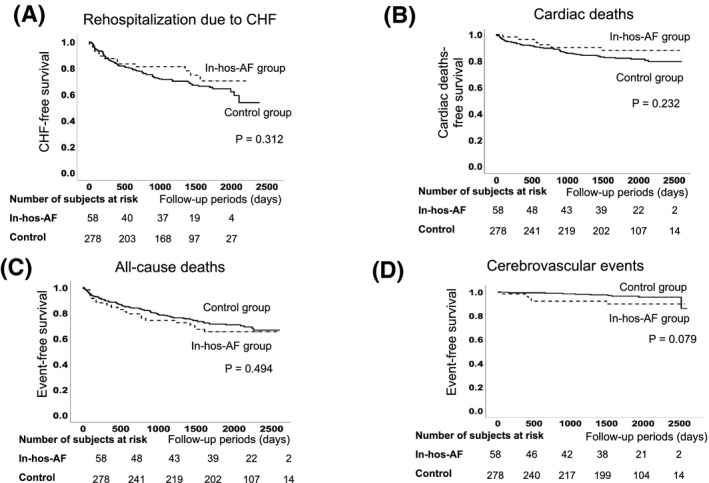
A, Kaplan‐Meier analysis for rehospitalization due to HF between the Control and the In‐hos‐AF groups. A total of 100 rehospitalizations due to worsening of HF were reported. B, Kaplan‐Meier analysis for cardiac death. The modes of cardiac death were heart failure (35 cases), sudden cardiac death (14 cases), ventricular tachyarrhythmia (six cases) and acute coronary syndrome (one case). The total numbers of cardiac deaths in each group was 50 in the Control group and six in the In‐hos‐AF group (*P* = 0.156). C, Kaplan‐Meier analysis for all‐cause death. The modes of all‐cause deaths other than cardiac death were pneumoniae (10 cases), malignant tumor (15 cases), infection (four cases), cerebrovascular infarction (four cases), cerebrovascular hemorrhage (three cases), old age (three cases), gastrointestinal hemorrhage (two cases), rupture of aortic aneurysm (two cases), suicide (one case), dehydration (one case), ileus (one case), liver cirrhosis (one cases), and unknown cause (one case).The total numbers of all‐cause deaths in each group was 84 in the Control group and 20 in the In‐hos‐AF group (*P* = 0.460). D, Kaplan‐Meier analysis for cerebrovascular events. The patient distributions in each group were 10 in the Control group and five in the In‐hos‐AF group (*P* = 0.187)

During the follow‐up period of 54 ± 24 months, the Kaplan‐Meier analysis demonstrated that there was no significant difference in rehospitalization due to HF, cardiac death, all‐cause deaths and cerebrovascular events between the Control and the In‐hos‐AF groups (Figure 3A–D).

These results indicate that new‐onset in‐hospital AF might have a limited impact on the clinical outcome after discharge. Therefore, the predisposing factor of the worse clinical outcomes was then investigated.

### 3.3 Prognostic impacts of new‐onset in‐hospital AF and AF after discharge on post discharge prognosis

In the Cox proportional hazard analysis (Table 2), the new‐onset in‐hospital AF was revealed to have no significant clinical impact on rehospitalization due to HF, cardiac deaths, all‐cause deaths and cerebrovascular events after discharge. In contrast, after adjusting for cofounding factors, AF development after discharge (47 patients in the Control and 24 in the In‐hos‐AF groups) was proven to be an independent risk factor for rehospitalization due to HF (HR 1.845, 95% CI: 1.019‐3.341, *P* = 0.043), cardiac deaths (HR 3.562, 95% CI: 1.311‐9.676, *P* = 0.013) and all‐cause deaths (HR 2.138, 95% CI: 1.127‐4.059, *P* = 0.020), but not cerebrovascular events (HR 0.409, 95% CI: 0.139‐1.207, *P* = 0.105).

**TABLE 2:** Cox proportional hazard model of rehospitalization because of HF, cardiac death, all‐cause death, and cerebrovascular events

	Unadjusted model			Adjusted model^a^		
	HR	95% CI	*P* value	HR	95% CI	*P* value
Rehospitalization because of CHF (100 events/n = 337)						
New onset in‐hospital AF	0.313	0.753‐2.417	.313			
AF after discharge	2.749	1.829‐4.132	**< .001**	1.845	1.019‐3.341	**.043**

	Unadjusted model			Adjusted model^b^		
	HR	95% CI	*P* value	HR	95% CI	*P* value
Cardiac death (56 events/n = 337)						
New onset in‐hospital AF	1.667	0.715‐3.889	.237			
AF after discharge	2.004	1.152‐3.486	**.014**	3.562	1.311‐9.676	**.013**

	Unadjusted model			Adjusted model^c^		
	HR	95% CI	*P* value	HR	95% CI	*P* value
All‐cause death (104 events/n = 337)						
New onset in‐hospital AF	0.844	0.518‐1.374	.495			
AF after discharge	1.668	1.096‐2.540	**.017**	2.138	1.127‐4.059	**.020**

	Unadjusted model			Adjusted model^d^		
	HR	95% CI	*P* value	HR	95% CI	*P* value
Cerebrovascular events (15 events/n = 337)						
New onset in‐hospital AF	0.395	0.135‐1.156	.09	0.409	0.139‐1.207	.105
AF after discharge	1.043	0.293‐3.713	.948			

Abbreviations: CI, confidence interval; HR, hazard ratio. The other abbreviations are the same as those in Table 1. *P* values in bold are statistically significant.

^a^ Adjusted model: Adjusted for age, BMI, BNP, EF, ischemic etiology, NYHA functional class, presence of CKD, usage of RAS inhibitor and DOAC.

^b^ Adjusted model: Adjusted for age, BMI, BNP, EF, presence of CKD and usage of RAS inhibitor.

^c^ Adjusted model: Adjusted for age, BMI, BNP, EF, ischemic etiology, NYHA functional class, presence of CKD, usage of RAS inhibitor, β‐blocker and diuretics.

^d^ Adjusted model: Adjusted for age and gender.

2

### 3.4 Sub‐analysis in the In‐hos‐AF group

**FIGURE 4:**
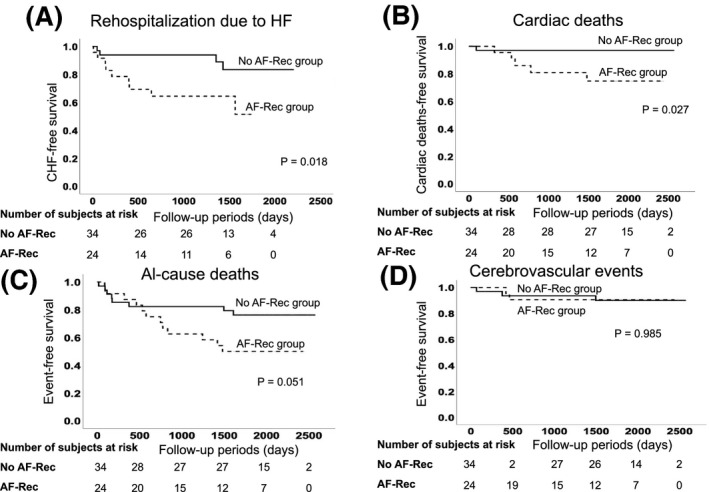
Comparison of clinical outcomes based on whether or not AF recurred in patients in the In‐hos‐AF group. A, Kaplan‐Meier analysis for rehospitalization due to HF between No AF recurrence and the AF recurrence groups. Rehospitalization due to HF was highly detected in the AF recurrence group than those in the No AF recurrence group (*P* = 0.018). B, Kaplan‐Meier analysis for cardiac death. More patients in the AF recurrence group suffered from cardiac deaths than those in the No AF recurrence group. C, Kaplan‐Meier analysis for all‐cause death. There was no statistical significance in all‐cause deaths between the groups (*P* = 0.051). D, Kaplan‐Meier analysis for cerebrovascular events. No statistical difference was found between the two groups (*P* = 0.985)

Next, the clinical implication of AF recurrence in the In‐hos‐AF group (n = 58) was investigated. AF recurred mean period of 527 ± 660 days after the first onset of AF during hospitalization. The In‐hos‐AF patients were assigned into two groups, as follows: the AF recurrence (AF‐Rec, n = 24) group and the No AF recurrence (No AF‐Rec, n = 34) group. Patients characteristics between the AF‐Rec group and the No AF‐Rec groups were shown in Table 3. The patients in the AF‐Rec group had a higher prevalence of CKD and were prescribed more diuretics, compared to those in the No AF‐Rec group, as shown in Table 3. As for AF characteristics, there were significantly more asymptomatic AF patients in the AF‐Rec group than those in the No AF‐Rec group. The duration between AF onset and termination in the No AF‐Rec group was shorter than that in the AF‐Rec group (1 ± 2 days vs 3 ± 4 days, *P* = 0.038). On the other hand, there was no statistical difference in the treatment of AF between the two groups. Then, clinical outcomes were compared using the Kaplan‐Meier analysis. The patients in the AF‐Rec group had a significantly higher risk of rehospitalization due to HF and cardiac deaths than those in the No AF‐Rec group (*P* = 0.018 and *P* = 0.027, respectively), as shown in Figure 4A,B. On the other hand, all‐cause deaths and cerebrovascular events did not differ between the two groups (*P* = 0.051 and *P* = 0.985 respectively; Figure 4C,D). In the multivariate Cox proportional hazard analysis, CKD was proven to be an independent predictor for AF recurrence (HR 3.076, 95% CI: 1.137‐8.321, *P* = 0.013), as shown in Table 4.

**TABLE 3:** Comparison of patient characteristics between groups AF‐Rec and No AF‐Rec

	No AF‐Rec group (n = 34)	AF‐Rec group (n = 24)	*P* value
Age (y)	63 ± 11	68 ± 17	0.149
Male (%)	74	67	0.580
BMI (kg/m^2^)	24.2 ± 3.6	22.7 ± 3.8	0.168
NYHA	1.9 ± 0.3	2.0 ± 0.4	0.241
CHA2DS2‐VASc score	4.1 ± 1.5	4.7 ± 1.6	0.135
Ischemic etiology (%)	35	25	0.413
Comorbidity			
HT (%)	79	96	0.076
DM (%)	44	50	0.665
DLp (%)	85	75	0.333
CKD (%)	50	79	**0.024**
Medication			
RAS inhibitor (%)	68	83	0.185
β‐blocker (%)	65	71	0.632
Diuretics (%)	56	88	**0.010**
Inotropic agents (%)	26	13	0.202
Anticoagulant (%)	50	63	0.355
DOAC (%)	3	4	0.805
VKA (%)	47	59	0.406
Lab data			
BNP	792 ± 1446	592 ± 585	0.601
Echo data			
LVEF (%)	54 ± 15	52 ± 15	0.571
LAVI (m^l^/m^2^)	32 ± 28	28 ± 20	0.598
AF characteristics			
Rapid AF (%)	44	54	0.459
Max HR during AF (bpm)	114 ± 35	116 ± 31	0.79
Symptomatic AF (%)	24	4	**0.027**
Duration from admission till AF onset (d)	7 ± 7	5 ± 6	0.213
Duration from AF onset till termination (days)	1 ± 2	3 ± 4	**0.038**
Treatment of AF			
None (%)	56	50	0.665
Landiolol (%)	15	8	0.472
Digitalis (%)	12	8	0.679
Verapamil (%)	0	4	0.237
Class I AAD (%)	12	8	0.679
Electric defibrillation (%)	18	29	0.088

Abbreviations: AAD, anti‐arrhythmic drugs; AF, atrial fibrillation; HR, heart rate; Max, maximum. The other abbreviations are the same as those in Table 1.

*P* values in bold are statistically significant.

**TABLE 4:** Cox proportional hazard model of AF recurrence after discharge

AF recurrence (24 events/n = 58)						
	Unadjusted model			Adjusted model		
	HR	95% CI	*P* value	HR	95% CI	*P* value
Asymptomatic AF	5.172	0.696‐38.452	.1.08			
CKD	3.201	1.190‐8.612	**.021**	3.076	1.137‐8.321	**.027**
Diuretics	3.396	1.102‐11.396	**.048**			
Duration from AF onset till termination	1.123	1.009‐1.249	**.034**			

Abbreviations: CI, confidence interval; HR, hazard ratio. The other abbreviations are the same as those in Table 3. 3

## 4 DISCUSSION

The major findings of the current study were as follows: (a) The clinical outcome post discharge of the In‐hos‐AF group was as good as that of the Control group in ADHF patients. (b) AF that developed after discharge was associated with rehospitalization due to HF, cardiac deaths, and all‐cause deaths. (c) In the In‐hos‐AF group, the patients in the AF‐Rec group showed higher risk of rehospitalization due to HF and cardiac deaths, compared with those in No AF‐Rec group. AF characteristics predisposing AF recurrence included asymptomatic and long‐duration AF. In addition, CKD was proven to be an independent predictor for AF recurrence.

### 4.1 New‐onset AF during hospitalization

In ADHF patients, the probable reasons for AF development were left ventricular dysfunction, excessive catecholamine release, acute hypoxia, sympathomimetic agents, and hypokalemia.^14^ While the AF‐inducing factors improved in the course of HF treatment, most cases of AF in the present study spontaneously terminated within a week during the mean period of hospital stay, 18 ± 10 days, in the In‐hos‐AF group. Given that almost all of the ADHF patients were compensated within the first 10 days after admission, most cases of in‐hospital new‐onset AF terminated at the early phase of admission. If HF is controlled and the AF‐inducing factors promoted by the ADHF do not further develop, the negative impact of new‐onset in‐hospital AF on cardiac function will be limited. Therefore, the clinical outcome of the patients in the In‐hos‐AF groups was as good as those of the Control group. A multicenter survey conducted in 24 European countries assessed the clinical impact of new‐onset in‐hospital AF, and concluded that patients with new‐onset AF had a longer intensive care unit stay and higher in‐hospital mortality, compared with a no‐AF group.^8^ Although the design of their study was similar to that of the present study, there were several differences in study outcomes as well as study design. The previous study did not evaluate the clinical outcome post discharge. In addition, although we evaluated the rehospitalization due to HF, cardiac deaths, all‐cause deaths as well as cerebrovascular events, the previous study included all‐cause death only. Therefore, it is difficult to directly compare the results of the current study with those of the previous study, because we placed importance on the prognosis not during hospital stay (the previous study), but after discharge in the present study.

### 4.2 AF development after discharge

It is well‐known that the patients who developed AF after discharge has been reported to be associated with worse prognosis. According to the Candesartan in Heart Failure: Assessment of Reduction in Mortality and Morbidity (CHARM) program, 6.15% of their study patients developed new AF.^15^ From the data of Japanese symptomatic HF patients (Chronic Heart Failure Analysis and Registry in the Tohoku District‐2 (CHART‐2) Study), 106 (3.6%) developed new AF, which was associated with an increased risk of cardiac death and admission because of HF.^16^ In the present study, we proved that the patients in the In‐hos‐AF group could have a worsened prognosis because of the increase in rehospitalization due to HF and cardiac deaths if they encountered AF recurrence after discharge (Figure 4). Moreover, AF after discharge was proven to be an independent risk factor for rehospitalization due to HF, cardiac death and all‐cause mortality in the Cox multivariate regression analysis (Table 2). In addition, the characteristics of AF in patients who had AF recurrence after discharge were asymptomatic and a longer duration till termination. These characteristics could promote atrial anatomical and electrical remodeling, resulting in AF recurrence afterwards and progression to persistent or chornic AF, which causes a significant increase in cardiac death. In fact, the EurObservational Research Programme‐Atrial Fibrillation (EORP‐AF) Pilot General Registry reported that asymptomatic AF patients were associated with a twofold higher mortality, compared with symptomatic patients.^17^ In the present study, Cox multivariate analysis revealed that CKD was an independent predictor for AF recurrence after discharge (Table 4). A prospective community‐based cohort study from Japan demonstrated a close bidirectional relationship between CKD and AF, which indicated that renal dysfunction increased the risk of new onset AF development, and AF increased the risk of CKD development.^18^ These results in the present study suggested that treatment for AF should be started immediately after the onset of AF after discharge was detected, because it resulted in worse prognosis in our HF patients.

Of note in the present study is that the discrepancy between the results of the clinical outcome and the prevalence of AF after discharge. Since the incidence of AF after discharge is significantly higher in the In‐hos‐AF group than in the Control group, the clinical outcome is expected to be better in the latter group. But the results were not significantly different. One of the possible reasons is that since AF has already begun to be treated in the In‐hos‐AF Group, even if it occurred after discharge from the hospital, AF tachycardia did not easily occur and it was difficult to lead to serious results. Actually, the incidence of hospitalization due to HF and the cardiac death in the In‐hos‐AF group tended to be greater than that of the Control group (58% vs 38%, *P* = 0.098 and 29% vs 21%, *P* = 0.457, respectively), which also supports this possibility.

Although it is difficult to clearly predict AF recurrence in patients in the In‐hos‐AF group, it is quite important to recognize that the patients’ prognosis might worsen after AF recurrence. This knowledge will bring the tangible benefit to some patients, not for all the patients, because there will be several events in the post discharge period that may affect outcome that was not studied.

### 4.3 Clinical implications

In the present study, most of the new‐onset AF in ADHF patients occurred within 7 days after admission, and terminated spontaneously. The post discharge clinical outcome was as good as those who had no AF episode unless AF recurred post discharge. In contrast, the prognosis of those who had AF development post discharge was poor. However, in many cases, it took a relatively long observation period of 527 days on average until recurrence. Therefore, treatments such as AAD, catheter ablation and anticoagulant might remain pending until AF recurrence. On the other hand, when AF development is detected after discharge from the hospital, aggressive treatment should be encouraged to avoid adverse cardiovascular events. In particular, the patients who had CKD were considered to be high risk for AF recurrence after discharge. In addition, as AF characteristics predisposing AF recurrence afterwards, asymptomatic and sustained AF should be paid attention.

### 4.4 Limitations

The present study has several limitations. First, as a prospective cohort study of a single center with a relatively small number of patients, the results may not be representative of the general population and may render the study underpowered to detect the differences. Second, the present study included only variables during hospitalization for decompensated HF, and we did not take into consideration changes in medical parameters post discharge which may affect the clinical outcome. Third, in the present study, there was a difference in the detection sensitivity of AF occurrence and symptoms between inpatients and outpatients. AF occurrence and the symptoms could be more likely to be detected in patients during hospitalization, compared with those after discharge. Fourth, in the present study, the small number of cerebrovascular events could be a reason why differences in cerebrovascular events were not detected between the groups, and might underpower the present study. Therefore, the present results should be viewed as preliminary, and further studies with a larger population are needed.

## 5 CONCLUSION

A new‐onset in‐hospital AF in ADHF subjects showed a spontaneous termination in most cases, and the clinical outcome was being as good as those who had no AF history. Therefore, treatment for new‐onset in‐hospital AF might be postponed until AF recurrence. On the other hand, in cases of AF recurrence after discharge, aggressive management might be recommended to prevent a worse prognosis.

## DISCLOSURES

The authors declare no Conflict of Interests for this article. The protocol for this research project has been approved by a suitably constituted Ethics Committee of the institution (No. 1808 on October 18th, 2013), and it conforms to the provisions of the Declaration of Helsinki. Committee of Fukushima Medical University, Approval No. 1808. All informed consent was obtained from the subjects.

## Footnotes

1 *P* values in bold are statistically significant.

2 *P* values in bold are statistically significant.

3 *P* values in bold are statistically significant.
